# Effect of the coverage of rapid tests for syphilis in primary care on the syphilis in pregnancy in Brazil

**DOI:** 10.11606/s1518-8787.2021055003264

**Published:** 2021-11-23

**Authors:** Angelo Giuseppe Roncalli, Tatyana Maria Silva de Souza Rosendo, Marquiony Marques dos Santos, Ana Karla Bezerra Lopes, Kenio Costa de Lima

**Affiliations:** I Universidade Federal do Rio Grande do Norte Centro de Ciências da Saúde Natal RN Brasils Universidade Federal do Rio Grande do Norte. Centro de Ciências da Saúde. Programa de Pós-Graduação em Saúde Coletiva. Natal, RN, Brasils; II Universidade Federal do Rio Grande do Norte Centro de Ciências da Saúde Departamento de Saúde Coletiva Natal RN Brasil Universidade Federal do Rio Grande do Norte. Centro de Ciências da Saúde. Departamento de Saúde Coletiva. Natal, RN, Brasil; III Universidade Federal do Rio Grande do Norte Laboratório de Inovação Tecnológica em Saúde Natal RN Brasil Universidade Federal do Rio Grande do Norte. Laboratório de Inovação Tecnológica em Saúde. Natal, RN, Brasil; IV Universidade Federal do Rio Grande do Norte Programa de Pós-Graduação em Ciências da Saúde Natal RN Brasil Universidade Federal do Rio Grande do Norte. Programa de Pós-Graduação em Ciências da Saúde. Natal, RN, Brasil

**Keywords:** Syphilis, epidemiology, Syphilis, Congenital, Syphilis Serodiagnosis, Prenatal Diagnosis, Health Services Coverage, Primary Health Care

## Abstract

**OBJECTIVE:**

To analyze the effect of rapid tests coverage in Primary Care on syphilis detection rate in pregnant women in Brazil, in municipalities with more than 100,000 inhabitants.

**METHODS:**

The dependent variable was the syphilis detection rate in pregnant women between 2012 and 2018. As the main independent variables, the methods for measuring the coverage of rapid tests for syphilis in Primary Care were used and, as adjustment variables, some indicators of health services and socioeconomic. We opted for a linear regression model for panel data (*panel data analysis*), considering the municipality as the unit of analysis and the year as the time variable.

**RESULTS:**

From the results of the final model, we can infer that, for a given municipality, as the rate of rapid tests increases by one point for every thousand live births, the detection rate of syphilis in pregnant women increases by an average of 0.02 cases per thousand live births (p < 0.001). This value is adjusted for Family Health coverage, proportion of health facilities per inhabitant, *per capita* expenditure on health and the Human Development Index.

**CONCLUSIONS:**

There was a substantial improvement in the amount of rapid tests available, as well as a significant increase in the number of tests performed in pregnant women, which predicts an increase in syphilis rates in pregnant women. However, a worrying hypothesis is that the number of tests performed on pregnant women during the analyzed period may have been insufficient to detect the progress of the epidemic in this population.

## INTRODUCTION

Syphilis is a sexually transmitted disease that, despite having a well-known etiology and treatment, has challenged health professionals due to its versatility in the clinical course^1^. Developed countries, such as Canada, the United States and those from Western Europe, which had their incidence of cases reduced in the 1980s and 1990s, showed an increase from the 2000s^2,3^. In the United States, the number of cases increased by 81% from 2014 to 2018, reaching the highest levels in the last 20 years^4^. Aspects such as HIV co-infection, behavioral changes after greater availability of effective antiretroviral treatment, and the use of internet dating apps have increased the complexity of controlling the disease^2^.

In Brazil, there has been an increase in registered cases of syphilis in recent years. When comparing a historical series from 2010 to 2018, the detection rate of acquired syphilis increased from 2.0 to 76.0 per 100,000 inhabitants, an increase of 3800%. An ascending behavior was also verified in the syphilis detection rates in pregnancy and in the incidence of congenital syphilis for the same period, reaching in 2018 estimates of 21.4 per thousand live births and nine per thousand live births, respectively^5^. Among the factors that may explain the increase in syphilis cases in pregnant women in Brazil are the improvement in the cases notification^6^, but also the low quality of prenatal care^7^ and the low proportion of adequate treatment for pregnant women and their partners. These aspects point to the need to improve the organization of services, intensifying serological testing and early treatment in pregnant women^8^.

In view of the worrying scenario of the disease in Brazil, nationwide strategies were implemented for its control, among which the project *“Sífilis Não”* stands out. This is a project aimed at one hundred cities with more than 100,000 inhabitants, distributed in all Brazilian regions (North, Northeast, Southeast, South and Center-West), which represent 57.7% of cases and approximately one third population, being considered priority cities to face the disease^9^. In addition to this initiative, other strategies are added, such as the purchase of diagnostic supplies (rapid tests) and treatment (penicillin), centralized by the Ministry of Health, in addition to carrying out a national prevention campaign and equipping situation rooms in priority municipalities of the project^5^.

In cities with more than 100,000 inhabitants in Brazil, priority or not of the *“Sífilis Não”* project, syphilis detection rates in pregnancy have grown in all regions of the country, with average annual increases ranging from 13.44% to 30.78% in the period between 2007 and 2017. There was an increase in cases in pregnant women in all the cities studied, with higher rates in those considered to be priorities in the South and Southeast regions of the country^10^.

Rapid tests (RT) enable the early diagnosis of the disease and, therefore, it is a good strategy for coping with the disease^11^. Between 2011 and 2017, in Brazil, the availability of rapid tests increased from 31,500 to 9,090,650^12^. A study carried out by Figueiredo et al.^13^ showed that Primary Care teams that performed rapid tests significantly expanded the identification and notification of cases in pregnant women, enabling timely prenatal care. This finding demonstrates the need to expand the availability and application of rapid tests for proper diagnosis and coping with the disease in pregnant women in the country.

In addition to the possible relationship between the increased availability of rapid tests and the increase in the number of syphilis cases in pregnant women, it is important to take into account other social, behavioral and health care factors that may be related to the increase in rates^14^. Therefore, the objective of this study is to analyze the effect of coverage of rapid tests in Primary Care on the detection rate of syphilis in pregnant women in Brazil, considering socioeconomic and health care contextual factors in municipalities with more than 100,000 inhabitants in Brazil.

## METHODS

### Type of Study

This is an ecological study, whose unit of aggregation were the Brazilian municipalities with more than 100,000 inhabitants.

### Data Source

Syphilis data in pregnant women were obtained from its main source, the Notifiable Diseases Information System (SINAN, from the Portuguese acronym). The socioeconomic data originated from the Atlas of Human Development in Brazil, whose indicators are calculated by UNDP Brazil and João Pinheiro Foundation, based on data from the decennial demographic censuses carried out by the Brazilian Institute of Geography and Statistics (IBGE). Indicators related to health services were obtained by consulting Datasus, through its tabulation interface, TabNet. The database of the Access and Quality Improvement in Primary Care Program (PMAQ, from the Portuguese acronym) was also used.

### Study Population

The data collected refer to municipalities with more than 100,000 inhabitants, as the inclusion of cities with a smaller population size would generate very fluctuating rates, as a single case significantly affects the population index. In addition, data from 2018 show that these 287 municipalities, focused on by the *“Sífilis Não”* project, concentrate 74% of syphilis cases in pregnancy and congenital and 73% of the records of the acquired disease.

### Variables

The Box illustrates the variables used in the study, their description, calculation method and respective sources, with the dependent variable being the syphilis detection rate in pregnancy from 2012 to 2018, obtained from SINAN.

**Box:** Variables used in the study, with their descriptions and sources.

Variable	Description and calculation method	Sources
Syphilis detection rate in pregnancy per thousand live births	Total syphilis cases detected in pregnant women per year of diagnosis / Total live births in the same year	SINAN/SINASC (www.datasus.gov.br)
Rapid tests in Primary Care referred in the PMAQ-AB	Number of health facilities that reported having a rapid test for syphilis always available / Total health facilities in the municipality x 100.	PMAQ (https://aps.saude.gov.br/ape/pmaq)
Rapid testing procedures for syphilis in pregnant women versus live births	Total rapid test procedures for syphilis in pregnant women / live births in the same period x ten thousand	SIA/IBGE (www.datasus.gov.br)
Coverage of the Family Health Strategy	Population coverage estimated by the Family Health Strategy teams, given by the percentage of the population covered by these teams in relation to the population estimate	E-Gestor AB (https://egestorab.saude.gov.br)
Primary Care Coverage	Estimated population coverage of Primary Care, given by the percentage of the population covered by teams from the Family Health Strategy and by teams of equivalent traditional Primary Care, parameterized in relation to the population estimate	E-Gestor (https://egestorab.saude.gov.br)
Primary Health Care Facilities per inhabitant	Number of Primary Health Care Facilities / Population in the same place and year x ten thousand	CNES (www.datasus.gov.br)
Health expenditures per inhabitant		
Municipal Human Development Index (HDI)	Geometric mean of the Income, Education and Longevity indices, with equal weights. Ranges from 0 to 1.	Atlas of Human Development (UNDP/IBGE) (www.atlasbrasil.org.br)
Gini index	Index that measures the degree of inequality according to *per capita* household income. It ranges from 0, when there is no inequality (the income of all individuals has the same value), to 1, when inequality is maximum (only one individual has all the income).	Atlas of Human Development (UNDP/IBGE) (www.atlasbrasil.org.br)

As main independent variables, two methods were used to measure the coverage of rapid tests for syphilis in Primary Care: in one of them, the database of the three cycles of the external evaluation of the Access and Quality Improvement Program (PMAQ) was used, years 2012, 2014 and 2017. The question “*I.11.1 – Rapid syphilis test always available*” was used, with possible answers “Yes and No”. For compatibility with the municipal database, the percentage of health units that answered “yes” to the question was calculated. In the second case, data from rapid testing procedures for syphilis offered to pregnant women in the primary care network, available in the SUS Outpatient Information System (SIA-SUS), were used. The query was made filtering by the approved amount of the procedure”*0214010082 - Rapid test for syphilis in pregnant women or father/partner*”, by municipality and by year, in the period between 2012 and 2018. The year 2012 was used as a starting point, because the policy of offering rapid tests in the primary care started in that year.

As adjustment variables, it was decided to incorporate health service and socioeconomic indicators. In the first case, coverage of the Family Health Strategy (ESF, from the Portuguese acronym), coverage of Primary Care, the proportion of Primary Care Health Facilities per inhabitant (per 10 thousand inhabitants) and health expenditures per inhabitant (in Brazilian reais) were used. As socioeconomic variables, it was decided to include a composite indicator that represents social conditions from the combination of indices on income, education and longevity, which is the HDI (Human Development Index), and an indicator of income inequality, the Gini index (Box).

### Data Analysis Strategies

Data obtained from different sources were converted to Stata format for analysis. For syphilis in pregnancy and for other health service indicators, data for the period between 2012 and 2018 are available. In the case of socioeconomic variables (HDI and Gini), the available data are only related to the 1991, 2000 and 2010 censuses. In this case, the values for the missing years were obtained by linear interpolation, as proposed by Rasella et al.^15^

Two analysis strategies were carried out, first, based on data on the availability of rapid tests by the PMAQ, classifying the municipalities into three categories: (a) available in all health facilities; (b) partially available and (c) not available in any. Based on this variable, the weighted averages of syphilis detection rates in pregnant women were calculated for each category, in the respective years. Since the data are census data, the averages were taken as true.

In the second strategy, the main independent variable was the proportion of rapid testing procedures for syphilis, offered to pregnant women in the primary care, using the basis of live births per thousand. The underlying hypothesis is that the higher the proportion of rapid tests, the higher the detection rate of syphilis in pregnancy in a given year and municipality. Since the variables are all continuous, we opted for a linear regression model for panel data (*panel data analysis*), considering the municipality as the unit of analysis and the year as the time variable. Regression for panel data is based on the following equation^16^.


$$Y_{it}=\alpha_{i}+\beta_{i}TRSAB_{it}+\beta_{n}X_{nit}+u_{it}$$

The outcome “Y” here means the detection rate of syphilis in pregnancy. The is the fixed effect for municipality *i* that captures all unobserved and time-invariant factors, “TRSAB” is the coverage of rapid tests for syphilis in Primary Care for a municipality *i* in year *t*, “X” represents the value of each of the adjustment variables for a municipality *i* in year *t* is the estimated error.

The fixed effects model was chosen because it is the most suitable for cases where all indicators vary over time. In addition, the Hausman test was applied to define between the fixed and random effects model.

## RESULTS

The first analysis carried out was based on the comparison of syphilis detection rates in pregnancy between municipalities with different categories of rapid test coverage, according to data from the three cycles of the PMAQ external evaluation (2011 to 2017).

**Figure 1:**
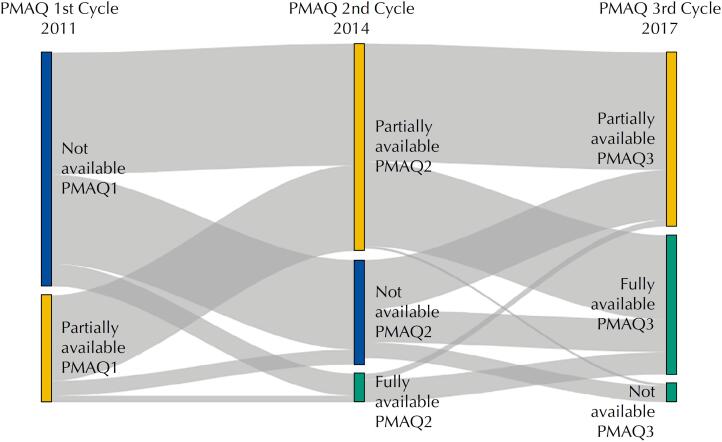
Availability of rapid test for syphilis in the primary healthcare according to the external assessment of the PMAQ in the 1^st^, 2^nd^ and 3^rd^ cycles (2011–2017).

Source: PMAQ. Brazil: 287 municipalities with more than 100,000 inhabitants.

A first important result can be seen in the flow chart in Figure 1, which shows an increasing coverage of rapid tests in Primary Care. In 2011, the highest percentage is of municipalities in the category “not available” and there is no municipality classified as “fully available”, a profile that has evolved in such a way that, in 2017, there are practically no more municipalities with the classification “not available” and most cities are between “partially available” and “fully available”.

**Figure 2:**
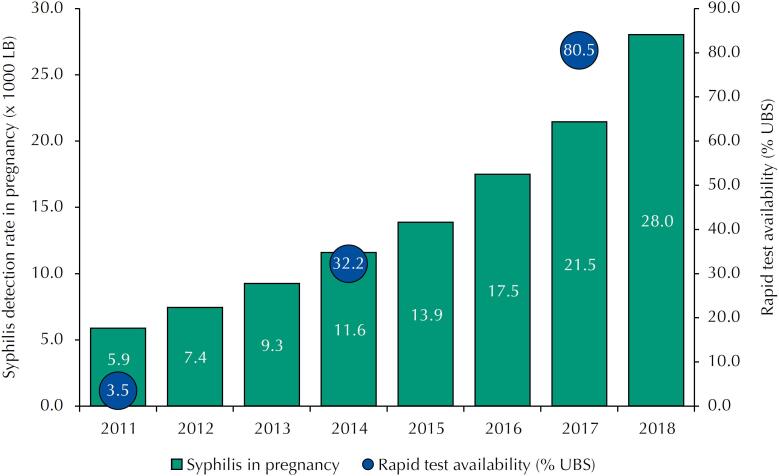
Evolution of the syphilis detection rate in pregnant women (per 1000 NV) and the proportion of primary health facilities with availability of a rapid syphilis test between 2011 and 2018 according to the PMAQ. Brazil: 287 municipalities with more than 100,000 inhabitants.

When analyzing the variable coverage of rapid tests in its original value (percentage), we can observe (Figure 2) that the averages of the proportions of health facilities with availability of rapid tests, grows from 3.5%, in 2011, to 80.5% in 2017, a concomitant growth with the syphilis detection rates in pregnancy.

Preliminary results already demonstrate a plausible relationship between the offer and application of rapid tests and the increase in syphilis detection rates in pregnant women, however, it is necessary to remember that other factors can influence the evolution of the data. In addition, the data provided by the external assessment of the PMAQ may contain biases, as this is self-reported information.

In this sense, a second analysis was performed, based on the total number of rapid tests for syphilis in pregnancy registered in the SIA-SUS, which made it possible to add other variables and verify the adjusted effect of the growth of these procedures in relation to syphilis in pregnant women.

**Figure 3:**
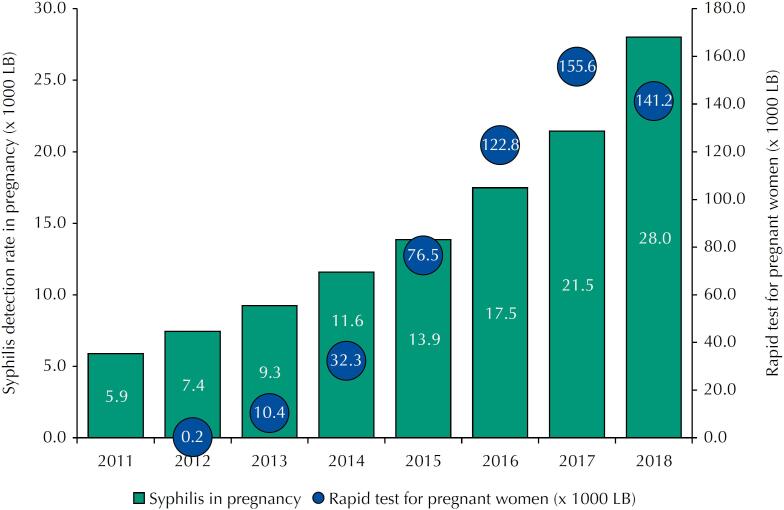
Syphilis detection rate in pregnant women and rapid test for syphilis in pregnant women (x 1,000 live births) between 2011 and 2018. Brazil: 287 municipalities with more than 100,000 inhabitants.

In Figure 3, we can observe a trend similar to the previous one regarding the PMAQ data, that is, the growth of rapid testing procedures for syphilis in pregnancy in the primary care network coincides with the growth of the detection rate of syphilis in pregnancy.

Although plausible, this result may have been influenced by other variables related to health services and the socioeconomic characteristics of the municipalities, so a linear regression was performed for panel data with a fixed effects model.

The Table shows the results of the regression, considering, as model 1, the inclusion of all variables that presented a “p” value less than 0.2 in the bivariate analysis and, in the final model, the variables that remained significant. From the results of the final model, it can be inferred that, for a given municipality, as the rate of rapid tests increases by one point for every thousand live births, the detection rate of syphilis in pregnant women increases by an average of 0.02 cases per thousand live births. This value is adjusted for Family Health coverage, proportion of primary health care facilities per inhabitant, *per capita* spending on health and HDI, with RHO being the intraclass correlation, it can be interpreted as the reason why 71.2% of the variance is due to differences between years.

**Table:** Panel data analysis, with fixed-effects regression model for the syphilis detection rate in pregnant women between 2012 and 2018.

Variable	Model 1			Final model		
	β	(IC95%)	p	β	(IC95%)	p
Rapid tests for pregnant women (x thousand live births)	0.018	0.016 a 0.021	< 0.001	0.004	0.002 a 0.007	< 0.001
FHS coverage (%)	0.343	0.295 a 0.391	< 0.001	0.070	0.027 a 0.112	0.001
Primary Care Coverage (%)	0.249	0.200 a 0.298	< 0.001	–	–	–
Number of UBS per 10,000 inhabitants	−3.255	−5.741 a -0.769	0.010	−2.924	−4.834 a -1.014	0.003
Health expenses per inhabitant (R$)	0.024	0.022 a 0.027	< 0.001	0.004	0.002 a 0.007	0.001
Municipal Human Development Index (HDI)	242.00	229.72 a 256.30	< 0.001	208.77	191.29 a 226.25	< 0.001
Gini index	−472.80	−507.80 a -437.80	< 0.001	–	–	–
Number of observations					1,992	
Number of groups					286	
R2 (within)					0.443	
R2 (between)					0.039	
Intraclass correlation (rho)					0.713	

## DISCUSSION

This is the first approximation in the literature to demonstrate the impact of rapid syphilis testing on syphilis rates in pregnant women from a multicenter study. There is a substantial growth in the supply of rapid syphilis tests in health facilities in the period from 2011 to 2017. This growth was accompanied by the rates of rapid tests performed in pregnant women and the rates of syphilis in pregnancy in health units. By controlling for confounding factors, it has been shown that increasing rapid syphilis testing in pregnant women significantly increases syphilis rates in pregnant women in the municipalities.

The growth in the supply of rapid syphilis tests in the country reflects the improvement of primary health care units (PHC) in the last decade. In general, Brazil has invested in the expansion of PHC services, mainly through the strengthening of Family Health Strategies (ESF) and increased coverage of care. Many of the ESF improvements stem from the concern to reduce several indicators of PHC-sensitive diseases, such as the fight against syphilis during pregnancy^17-19^.

The health care of women and children were the starting points for the improvements of the ESF, from national programs to reduce maternal, fetal and neonatal mortality, such as the provision of prenatal care in Brazil, which reached 98.7% of care for pregnant women in 2014, currently, no state has less than 90% of prenatal care. This improvement increases the ability of PHC to diagnose and treat diseases, especially during pregnancy, which could justify the considerable increase in rapid syphilis tests in recent years^20^.

In 2011 the Ministry of Health launched Ordinance No. 1,459, which institutes the Rede Cegonha program, with the objective of ensuring the right to reproductive planning and humanized care for pregnancy. Within the program, new tests financed by the federal government were launched, such as the implementation of rapid tests for syphilis, carried out during prenatal consultations^21^. Therefore, the set of improvements previously reported in the PHC, with the strengthening of the FHS and the constant remodeling of public health policies that seek to increase the system's capacity for diagnosis and treatment, as in the case of syphilis, influenced the considerable increase in rapid tests in the health care network.

This study showed that the growth in the performance of rapid tests in pregnant women in the cities significantly increased the number of records of syphilis cases in pregnant women, regardless of other factors, such as the number of health care facilities, increased coverage of the ESF or the HDI. The rapid test is part of the algorithm for detecting syphilis during pregnancy in PHC, mainly because it is easy to perform and low cost, and can be performed anywhere, including in campaigns to fight against the disease^22^. By demonstrating that the rapid test is an essential diagnostic screening tool for the early detection of syphilis in pregnant women, it increases its importance in decision-making to increase its funding.

A review conducted by Angel-Müller et al. ^23^ indicates that the *point of care testing* initiative is an effective strategy for screening syphilis during prenatal care, aiding the diagnosis in pregnant women. Although the aforementioned study does not indicate how much of an increase in the diagnostic success rate is credited to *screening* by the rapid test, its large-scale use for pregnant women in PHC may prove to be much more effective than other diagnostic strategies, mainly because the data obtained point to this relationship. The authors emphasize that at least 95% of pregnant women should undergo testing in the first prenatal visit.

In Brazil, the number of rapid tests for syphilis is determined by the clinical protocol of therapeutic guidelines, establishing that pregnant women must undergo three rapid tests: the first in the first trimester of pregnancy; the second at the beginning of the third trimester; and the third at the time of childbirth or abortion, regardless of previous exams^24^. However, we found a value much lower than that established by the protocols, reaching a number of rapid tests almost eight times lower than the minimum number for a single test per live birth.

The last national health survey found a coverage rate of syphilis tests in pregnant women of 64.8%^25^, another study using a hospital database showed that no region in Brazil reached 95% testing for syphilis in the first consultation of the prenatal. When verifying a second test, the maximum coverage value reached only 56%^20^. Thus, the effect of performing rapid tests to detect syphilis during pregnancy in PHC may be even greater than those found, which could indicate an even more evident importance of the use of rapid tests in these locations for detecting syphilis during the prenatal care.

The quality of prenatal care is one of the important factors for carrying out rapid tests in PHC, which is corroborated by an article developed by Mário et al.^26^ investigated the quality of prenatal care based on a national survey, pointing out that 80.6% of pregnant women were classified as having adequate prenatal care and, although the effect of the rapid test on the quality of prenatal care has not directly worked, the diagnosis of syphilis in PHC is part of the classification of an adequate prenatal care. Thus, we can hypothesize that the improvement in the quality of prenatal care in Brazilian cities may have increased the number of tests in pregnant women, mainly due to the strengthening of national programs to improve the health of pregnant women and protect the newborn.

As a limitation of this study, caution should be exercised when interpreting studies that use secondary data. In addition, the data provided by the external assessment of the PMAQ may contain biases, as this is self-reported information. In addition, other variables that were not tested could find different predictive effects in the relationship between the rapid test and the rates of syphilis in pregnant women, however, as this study used national databases, this is the information available that comes closest to a relationship between syphilis testing and the increase in syphilis in pregnancy in Brazil.

## CONCLUSIONS

It is intuitive to think that increasing the rapid test in PHC increases the ability to detect syphilis in pregnancy, which was proven by this study. There was a substantial improvement in the amount of rapid tests available, in addition to a significant increase in the number of tests performed in pregnant women, which predicts an increase in syphilis rates in pregnant women.

However, given the results presented, we can suggest that the number of tests performed on pregnant women in the period analyzed was insufficient to detect the progress of the epidemic in this population, mainly due to the presence of a stabilization in the number of rapid tests performed and the absence of a plateau of syphilis rates in pregnancy. This hypothesis is worrying because it may indicate that the lack of control of syphilis in pregnancy is, in part, due to the insufficient number of tests to detect existing cases in this population.

Therefore, investing in increasing the supply of rapid syphilis tests for pregnant women, in addition to subsidizing strategies that strengthen the increase in the number of these tests during prenatal care, is an important strategy to control the epidemic in the country, which could reduce rates of congenital syphilis. The development of new studies that seek to assess the cost-effectiveness of this measure at the local level, compared to other strategies, could expand the criteria for using a rapid test in the pregnant population.
