# Supplementary material for: Effect of the coverage of rapid tests for syphilis in primary care on the syphilis in pregnancy in Brazil
Source: Rev Saude Publica. 2021 Nov 23;55:94. doi: 10.11606/s1518-8787.2021055003264 (PMC8621623; doi:10.11606/s1518-8787.2021055003264)
Supplement: Translation

# Translation: Efeito da cobertura de testes rápidos na atenção básica sobre a sífilis em gestantes no Brasil

**Authors:** Angelo Giuseppe Roncalli, Tatyana Maria Silva de Souza Rosendo, Marquiony Marques dos Santos, Ana Karla Bezerra Lopes, Kenio Costa de Lima

**Affiliations:** I Natal RN Brasil Universidade Federal do Rio Grande do Norte. Centro de Ciências da Saúde. Programa de Pós-Graduação em Saúde Coletiva. Natal, RN, Brasil; II Natal RN Brasil Universidade Federal do Rio Grande do Norte. Centro de Ciências da Saúde. Departamento de Saúde Coletiva. Natal, RN, Brasil; III Natal RN Brasil Universidade Federal do Rio Grande do Norte. Laboratório de Inovação Tecnológica em Saúde. Natal, RN, Brasil; IV Natal RN Brasil Universidade Federal do Rio Grande do Norte. Programa de Pós-Graduação em Ciências da Saúde. Natal, RN, Brasil

**Keywords:** Sífilis, epidemiologia, Sífilis Congênita, Sorodiagnóstico da Sífilis, Diagnóstico Pré-Natal, Cobertura de Serviços de Saúde, Atenção Primária à Saúde

## Abstract

**OBJETIVO:**

Analisar o efeito da cobertura de testes rápidos na Atenção Básica sobre a taxa de detecção de sífilis em gestantes no Brasil, nos municípios com mais de 100 mil habitantes.

**MÉTODOS:**

A variável dependente foi a taxa de detecção de sífilis em gestantes entre os anos de 2012 e 2018. Como variáveis independentes principais, foram utilizados os métodos de aferição da cobertura de testes rápidos para sífilis na Atenção Básica e, como variáveis de ajuste, alguns indicadores de serviços de saúde e socioeconômicos. Optou-se por um modelo de regressão linear para dados em painel (*panel data analysis*), considerando o município como unidade de análise e ano como variável de tempo.

**RESULTADOS:**

Pelos resultados do modelo final, pode-se inferir que, para um determinado município, à medida que a taxa de testes rápidos aumenta em um ponto para cada mil nascidos vivos, a taxa de detecção de sífilis em gestantes aumenta em média 0,02 casos por mil nascidos vivos (p < 0,001). Esse valor está ajustado para cobertura de Saúde da Família, proporção de UBS por habitante, gastos *per capita* com saúde e Índice de Desenvolvimento Humano.

**CONCLUSÕES:**

Houve uma melhora substancial na quantidade de testes rápidos disponíveis, bem como, o aumento significativo de realização desses testes em gestantes, o que prediz o aumento das taxas de sífilis em gestantes. Contudo, uma hipótese preocupante é que a quantidade de testes realizados em gestantes no período analisado pode ter sido insuficiente para detectar o avanço da epidemia nessa população.

## INTRODUÇÃO

A sífilis é uma doença sexualmente transmissível que, apesar de possuir etiologia e tratamento notadamente conhecidos, tem desafiado os profissionais de saúde pela sua versatilidade no curso clínico da doença^1^. Países desenvolvidos, como Canadá, Estados Unidos e da Europa Ocidental, que tiveram sua incidência de casos reduzida nas décadas de 1980 e 1990, apresentaram aumento a partir dos anos 2000^2,3^. Nos Estados Unidos o número de casos aumentou em 81% no período de 2014 a 2018, atingindo os mais altos níveis dos últimos 20 anos^4^. Aspectos como a coinfecção pelo HIV, mudanças comportamentais após maior disponibilidade de tratamento antirretroviral eficaz e uso de aplicativos de encontro pela internet têm aumentado a complexidade para o controle da doença^2^.

No Brasil, observa-se um aumento nos casos registrados de sífilis nos últimos anos. Na comparação em uma série histórica, entre 2010 e 2018, a taxa de detecção de sífilis adquirida passou de 2,0 para 76,0 por 100.000 habitantes, um aumento de 3.800%. Comportamento ascendente também foi verificado nas taxas de detecção da sífilis em gestantes e na incidência de sífilis congênita para o mesmo período, atingindo em 2018 estimativas de 21,4 por mil nascidos vivos e nove por mil nascidos vivos, respectivamente^5^. Dentre os fatores que podem explicar o aumento dos casos de sífilis em gestantes no Brasil estão a melhora na notificação dos casos^6^, mas também a baixa qualidade do pré-natal^7^ e a baixa proporção de tratamento adequado da gestante e de suas parcerias. Tais aspectos apontam a necessidade de melhorar a organização dos serviços, intensificar a testagem sorológica e o tratamento precoce em gestantes^8^.

Diante do cenário preocupante da doença no Brasil, estratégias de abrangência nacional foram implementadas para seu controle, dentre as quais destaca-se o projeto “Sífilis Não”. Trata-se de um projeto direcionado a cem municípios com mais de 100.00 habitantes, distribuídos em todas as regiões brasileiras (Norte, Nordeste, Sudeste, Sul e Centro-Oeste), que representam 57,7% dos casos e, aproximadamente, um terço da população, sendo considerados municípios prioritários para o enfrentamento da doença^9^. Somam-se a essa iniciativa outras estratégias, como a compra de insumos de diagnóstico (testes rápidos) e tratamento (penicilina), centralizada pelo Ministério da Saúde, além da realização de campanha nacional de prevenção e da instrumentalização de salas de situação nos municípios prioritários do projeto^5^.

Nas cidades com mais de 100.000 habitantes do Brasil, prioritárias ou não do projeto “Sífilis Não”, as taxas de detecção de sífilis em gestantes têm crescido em todas as regiões do país, com incrementos médios anuais variando de 13,44% a 30,78% no período entre 2007 e 2017. Constatou-se aumento de casos em gestantes em todos os municípios estudados, com índices maiores naqueles considerados prioritários nas regiões Sul e Sudeste do país^10^.

Os testes rápidos (TR) possibilitam o diagnóstico precoce da doença e, portanto, mostra-se uma boa estratégia de enfrentamento da doença^11^. Entre 2011 e 2017, no Brasil, a disponibilização de testes rápidos saltou de 31.500 para 9.090.650^12^. Estudo realizado por Figueiredo e colaboradores^13^ mostrou que as equipes da Atenção Básica que realizavam os testes rápidos ampliaram de forma significativa a identificação e notificação de casos em gestantes, possibilitando o cuidado oportuno no pré-natal. Tal achado demonstra a necessidade de ampliação da disponibilidade e aplicação de testes rápidos para o devido diagnóstico e enfrentamento da doença em gestantes no país.

Além da possível relação entre o aumento da disponibilidade de testes rápidos e aumento do número de casos de sífilis em gestantes, é importante que se leve em consideração outros fatores sociais, comportamentais e de assistência à saúde que podem estar relacionados ao aumento das taxas^14^. Diante disso, o objetivo deste estudo é analisar o efeito da cobertura de testes rápidos na Atenção Básica sobre a taxa de detecção de sífilis em gestantes no Brasil, considerando fatores socioeconômicos e de assistência à saúde contextuais nos municípios com mais de 100 mil habitantes do Brasil.

## MÉTODOS

### Tipo de Estudo

Trata-se de um estudo ecológico, cuja unidade de agregação foram os municípios brasileiros com mais de 100 mil habitantes.

### Fonte dos Dados

Os dados de sífilis em gestantes foram obtidos a partir de sua principal fonte, o Sistema de Informações de Agravos de Notificação (SINAN). Os dados socioeconômicos se originaram do Atlas de Desenvolvimento Humano do Brasil, cujos indicadores são calculados pelo PNUD Brasil e Fundação João Pinheiro, a partir dos dados dos censos demográficos decenais realizados pelo Instituto Brasileiro de Geografia e Estatística (IBGE). Indicadores relativos aos serviços de saúde foram obtidos a partir de consulta ao Datasus, por meio de sua interface de tabulação, o TabNet. Foi utilizada, ainda, a base de dados do Programa de Melhoria do Acesso e da Qualidade da Atenção Básica (PMAQ).

### População do Estudo

Os dados coletados referem-se aos municípios com mais de 100 mil habitantes, pois a inclusão de cidades com porte populacional menor geraria taxas muito flutuantes, já que um único caso afeta de modo significativo o índice populacional. Além disso, dados de 2018 apontam que esses 287 municípios, focalizados pelo projeto “Sífilis Não”, concentram 74% dos casos de sífilis em gestantes e congênita e 73% dos registros da doença adquirida.

### Variáveis

O Quadro 1 ilustra as variáveis utilizadas no estudo, sua descrição, forma de cálculo e respectivas fontes, sendo que a variável dependente é a taxa de detecção de sífilis em gestantes nos anos de 2012 a 2018, obtida junto ao SINAN.

**Quadro 1:** Variáveis utilizadas no estudo, com as respectivas descrições e fontes.

Variável	Descrição e forma de cálculo	Fontes
Taxa de detecção de sífilis em gestantes por mil nascidos vivos	Total de casos de sífilis detectados em gestantes por ano de diagnóstico / Total de nascidos vivos no mesmo ano	SINAN / SINASC (www.datasus.gov.br)
Testes rápidos na Atenção Básica referido no PMAQ-AB	Número de unidades de saúde que relataram ter teste rápido para sífilis sempre disponível / Total de unidades básicas do município x cem.	PMAQ (https://aps.saude.gov.br/ape/pmaq)
Procedimentos de testes rápidos para sífilis em gestantes em relação aos nascidos vivos	Total de procedimentos de testes rápidos para sífilis em gestantes / nascidos vivos no mesmo período x dez mil	SIA / IBGE (www.datasus.gov.br)
Cobertura da Estratégia Saúde da Família	Cobertura populacional estimada pelas equipes da Estratégia Saúde da Família, dada pelo percentual da população coberta por essas equipes em relação à estimativa populacional	E-Gestor AB (https://egestorab.saude.gov.br)
Cobertura da Atenção Básica	Cobertura populacional estimada na Atenção Básica, dada pelo percentual da população coberta por equipes da Estratégia Saúde da Família e por equipes de Atenção Básica tradicional equivalentes e parametrizadas em relação à estimativa populacional	E-Gestor (https://egestorab.saude.gov.br)
Unidades Básicas de Saúde por habitante	Número de Unidades Básicas de Saúde / População no mesmo local e ano x dez mil	CNES (www.datasus.gov.br)
Gastos com saúde por habitante		
Índice de Desenvolvimento Humano (IDH) Municipal	Média geométrica dos índices das dimensões Renda, Educação e Longevidade, com pesos iguais. Varia de 0 a 1.	Atlas de Desenvolvimento Humano (PNUD / IBGE) (www.atlasbrasil.org.br)
Índice de Gini	Índice que mede o grau de desigualdade segundo a renda domiciliar *per capita*. Varia de 0, quando não há desigualdade (a renda de todos os indivíduos tem o mesmo valor), a 1, quando a desigualdade é máxima (apenas um indivíduo detém toda a renda).	Atlas de Desenvolvimento Humano (PNUD / IBGE) (www.atlasbrasil.org.br)

Como variáveis independentes principais, foram utilizados dois métodos de aferição da cobertura de teste rápidos para sífilis na Atenção Básica: em um deles, foi utilizada a base de dados dos três ciclos da avaliação externa do Programa de Melhoria do Acesso e da Qualidade (PMAQ), anos de 2012, 2014 e 2017. Foi utilizada a questão “*I.11.1 – Teste rápido de sífilis sempre disponível*”, com as respostas possíveis “Sim e Não”. Para a compatibilização com o banco de dados de base municipal, foi calculado o percentual de unidades de saúde que responderam “sim” à questão. No segundo caso, foram utilizados os dados de procedimentos de testes rápidos para sífilis ofertados à gestante na rede básica, disponível no Sistema de Informações Ambulatoriais do SUS (SIA-SUS). Foi feita a consulta filtrando pela quantidade aprovada do procedimento “*0214010082 – Teste rápido para sífilis na gestante ou pai/parceiro*”, por município e por ano, no período entre 2012 e 2018. O ano de 2012 foi utilizado como ponto inicial, porque a política de oferta de testes rápidos na rede básica iniciou-se nesse ano.

Como variáveis de ajuste, decidiu-se pela incorporação de indicadores de serviços de saúde e socioeconômicos. No primeiro caso, foi utilizada a cobertura da Estratégia Saúde da Família (ESF), a cobertura da Atenção Básica, a proporção de Unidades Básicas de Saúde por habitante (por 10 mil habitantes) e os gastos com saúde por habitante (em reais). Como variáveis socioeconômicas, decidiu-se pela inclusão de um indicador composto que representa as condições sociais a partir da junção de índices sobre renda, escolaridade e longevidade, que é o IDH (Índice de Desenvolvimento Humano), e um indicador de desigualdade de renda, o índice de Gini (Quadro 1).

### Estratégias de Análise dos Dados

Os dados obtidos de diferentes fontes foram convertidos para o formato Stata para a realização das análises. Para as variáveis de sífilis em gestantes e para os outros indicadores de serviços de saúde, os dados para o período entre 2012 e 2018 estão disponíveis. No caso das variáveis socioeconômicas (IDH e Gini) os dados disponíveis são apenas relativos aos censos de 1991, 2000 e 2010. Nesse caso, os valores para os anos faltantes foram obtidos por interpolação linear, de acordo com proposta de Rasella et al.^15^

Foram realizadas duas estratégias de análise, primeiro tendo como base os dados de disponibilidade de testes rápidos pelo PMAQ, classificando os municípios em três categorias: (a) disponível em todas as unidades de saúde; (b) parcialmente disponível e (c) não disponível em nenhuma. A partir dessa variável, calculou-se as médias ponderadas das taxas de detecção de sífilis em gestantes para cada uma das categorias, nos respectivos anos. Uma vez que os dados são censitários, as médias foram tomadas como verdadeiras.

Na segunda estratégia, a variável independente principal foi a proporção de procedimentos de testes rápidos para sífilis, ofertados para gestantes na rede básica, utilizando-se a base de nascidos vivos por mil. A hipótese subjacente é que quanto maior a proporção de testes rápidos, maior será a taxa de detecção de sífilis em gestantes em um dado ano e município. Uma vez que as variáveis são todas de natureza contínua, optou-se por um modelo de regressão linear para dados em painel (*panel data analysis*), considerando o município como unidade de análise e ano como variável de tempo. A regressão para dados em painel baseia-se na equação a seguir^16^.


$$Y_{it}=\alpha_{i}+\beta_{i}TRSAB_{it}+\beta_{n}X_{nit}+u_{it}$$

O desfecho “Y” significa, aqui, a taxa de detecção de sífilis em gestantes. O é o efeito fixo para o município *i* que captura todos os fatores não observados e invariantes no tempo, “TRSAB” é a cobertura de testes rápidos para sífilis na Atenção Básica para um município *i* no ano *t*, “X” representa o valor de cada uma das variáveis de ajuste para um município *i* no ano *t* e é o erro estimado.

O modelo de efeitos fixos foi escolhido por ser o mais adequado para os casos em que todos os indicadores variam ao longo tempo. Além disso, foi aplicado o teste de Hausman para a definição entre o modelo de efeitos fixos ou aleatórios.

## RESULTADOS

A primeira análise realizada tomou como base a comparação das taxas de detecção de sífilis em gestantes entre os municípios com diferentes categorias de cobertura de testes rápidos, segundo dados dos três ciclos da avaliação externa do PMAQ (2011 a 2017).

Um primeiro resultado importante pode ser observado no gráfico de fluxos da Figura 1, que mostra uma cobertura crescente dos testes rápidos na Atenção Básica. Em 2011, o maior percentual é de municípios na categoria “não disponível” e não há nenhum município classificado como “totalmente disponível”, perfil que evolui de tal modo que, em 2017, praticamente não se verifica mais municípios com a classificação “não disponível” e grande parte das cidades está entre “parcialmente disponível” e “totalmente disponível”.

**Figura 1:** Disponibilidade de teste rápido para sífilis na rede básica de saúde de acordo com a avaliação externa do PMAQ no 1°, 2° e 3° ciclos (2011–2017).

Fonte: PMAQ. Brasil: 287 municípios com mais de 100.000 habitantes.

Quando se analisa a variável de cobertura de testes rápidos em seu valor original (percentual), podemos observar (Figura 2) que as médias das proporções de unidades de saúde com disponibilidade de testes rápidos, cresce de 3,5%, em 2011, para 80,5%, em 2017, crescimento concomitante ao das taxas de detecção de sífilis em gestantes.

**Figura 2:** Evolução da taxa de detecção de sífilis em gestantes (por 1000 NV) e a proporção de unidades básicas de saúde com disponibilidade de teste rápido para sífilis entre 2011 e 2018 segundo o PMAQ. Brasil: 287 municípios com mais de 100.000 habitantes.

Os resultados preliminares já demonstram plausível a relação entre a oferta e aplicação de testes rápidos e o aumento nas taxas de detecção de sífilis em gestantes, porém, é preciso lembrar que outros fatores podem influenciar a evolução dos dados. Além disso, os dados fornecidos pela avaliação externa do PMAQ podem conter vieses, uma vez que se trata de informação autorreferida.

Nesse sentido, foi realizada uma segunda análise, tendo como base o total de testes rápidos para sífilis em gestantes registrados no SIA-SUS, o que possibilitou acrescentar outras variáveis e verificar o efeito ajustado do crescimento desses procedimentos em relação à sífilis em gestantes.

Na Figura 3 podemos observar uma tendência similar à anterior sobre os dados do PMAQ, ou seja, o crescimento dos procedimentos de testes rápidos para sífilis em gestantes na rede básica é coincidente com o crescimento da sífilis em gestantes.

**Figura 3:** Taxa de detecção de sífilis em gestantes e teste rápido para sífilis na gestante (x 1.000 nascidos vivos) entre 2011 e 2018. Brasil: 287 municípios com mais de 100.000 habitantes.

Embora plausível, esse resultado pode ter sofrido interferência de outras variáveis relativas aos serviços de saúde e às características socioeconômicas dos municípios, por isso foi realizada uma regressão linear para dados em painel com modelo de efeitos fixos.

A Tabela 1 mostra os resultados da regressão, considerando, como modelo 1, a inclusão de todas as variáveis que apresentaram valor de “p” menor que 0,2 na análise bivariada e, no modelo final, as variáveis que permaneceram significativas. Pelos resultados do modelo final, pode-se inferir que, para um determinado município, à medida que a taxa de testes rápidos aumenta em um ponto para cada mil nascidos vivos, a taxa de detecção de sífilis em gestantes aumenta em média 0,02 casos por mil nascidos vivos. Esse valor está ajustado para cobertura de Saúde da Família, proporção de UBS por habitante, gastos *per capita* com saúde e IDH, sendo RHO a correlação intraclasse, pode-se interpretá-la como a razão para que 71,2% da variância se deva a diferenças entre os anos.

**Tabela 1:** Análise de dados em painel, com modelo de regressão para efeitos fixos para a taxa de detecção de sífilis em gestantes entre 2012 e 2018.

Variável	Modelo 1			Modelo Final		
	β	(IC95%)	p	β	(IC95%)	p
Testes rápidos para gestantes (x mil nascidos vivos)	0,018	0,016 a 0,021	< 0,001	0,004	0,002 a 0,007	< 0,001
Cobertura da ESF (%)	0,343	0,295 a 0,391	< 0,001	0,070	0,027 a 0,112	0,001
Cobertura da Atenção Básica (%)	0,249	0,200 a 0,298	< 0,001	–	–	–
Número de UBS por 10 mil habitantes	−3,255	−5,741 a -0,769	0,010	−2,924	−4,834 a -1,014	0,003
Gastos com saúde por habitante (R$)	0,024	0,022 a 0,027	< 0,001	0,004	0,002 a 0,007	0,001
Índice de Desenvolvimento Humano (IDH)	242,00	229,72 a 256,30	< 0,001	208,77	191,29 a 226,25	< 0,001
Índice de Gini	−472,80	−507,80 a -437,80	< 0,001	–	–	–
Número de observações					1.992	
Número de grupos					286	
R^2^ (dentro)					0,443	
R^2^ (entre)					0,039	
Correlação intraclasse (rho)					0,713	

## DISCUSSÃO

Esta é a primeira aproximação na literatura que demonstra o impacto do teste rápido da sífilis sobre as taxas de sífilis em gestantes a partir de um estudo multicêntrico. Existe um crescimento substancial da oferta de testes rápidos de sífilis nas unidades de saúde no período de 2011 a 2017. Esse crescimento foi acompanhado dos índices de realização dos testes rápidos em gestantes e das taxas de sífilis em gestantes nas unidades de saúde. Ao controlar os fatores de confusão, foi demonstrado que o aumento dos testes rápidos de sífilis em gestantes aumenta significativamente as taxas de sífilis em gestantes nos municípios.

O crescimento da oferta de testes rápidos da sífilis no país reflete na melhoria das unidades de atenção primária em saúde (APS) na última década. De maneira geral, o Brasil tem investido na ampliação de serviços da APS, principalmente através do fortalecimento das Estratégias de Saúde da Família (ESF) e aumento de cobertura no atendimento. Muitas das melhorias das ESF partem da preocupação em reduzir diversos indicadores de doenças sensíveis à APS, como o combate à sífilis na gestação^17-19^.

A atenção à saúde da mulher e da criança foram os pontos de partidas das melhorias da ESF, a partir de programas nacionais de redução de mortalidade materna, fetal e neonatal, como por exemplo, a realização de assistência ao pré-natal no Brasil, que atingiu 98,7% de atendimentos em gestantes no ano de 2014, atualmente, nenhum estado possui menos de 90% de assistência ao pré-natal. Essa melhoria aumenta a capacidade da APS em diagnosticar e tratar doenças, principalmente na gestação, o que poderia justificar o aumento considerável dos testes rápidos de sífilis nos últimos anos^20^.

Em 2011 o Ministério da Saúde lança a Portaria n° 1.459, que institui o programa Rede Cegonha, com objetivo de assegurar o direito ao planejamento reprodutivo e à atenção humanizada à gravidez. Dentro do programa, foi lançado novos exames financiados pelo governo federal, como a implantação dos testes rápidos da sífilis, realizados durante a consulta pré-natal^21^. Portanto, o conjunto de melhorias relatados anteriormente na APS, com o fortalecimento das ESF e as constantes remodelagens das políticas públicas de saúde que buscam aumentar a capacidade de diagnóstico e tratamento do sistema, como no caso da sífilis, influenciaram o aumento considerável dos testes rápidos na rede de atenção à saúde.

Este estudo mostrou que o crescimento da realização dos testes rápidos em gestantes nos municípios aumentou significativamente o número registros dos casos de sífilis em gestantes, independente de outros fatores, como o número de UBS, aumento da cobertura da ESF ou do IDHM. O teste rápido faz parte do algoritmo para detecção de sífilis na gestação na APS, principalmente por ser de fácil execução e de baixo custo, podendo ser realizado em qualquer lugar, inclusive em campanhas de combate à doença^22^. Ao demonstrar que a realização do teste rápido é uma ferramenta de triagem diagnóstica fundamental para a detecção precoce de sífilis em gestantes, eleva sua importância na tomada de decisão para aumentar seu financiamento.

Uma revisão conduzida por Angel-Müller e colaboradores^23^, indicam que a iniciativa de *point of care testing* é uma estratégia eficaz para triagem da sífilis durante o pré-natal, auxiliando o diagnóstico em gestantes. Embora o referido estudo não aponte o quanto de incremento da taxa de sucesso de diagnóstico é credenciado ao *screening* pelo teste rápido, o seu uso em larga escala para gestantes na APS pode se mostrar muito mais efetivo do que outras estratégias de diagnóstico, principalmente porque os dados obtidos apontam para essa relação. Os autores reforçam que ao menos 95% das mulheres grávidas devem realizar a testagem na primeira consulta ao pré-natal.

No Brasil, o número de testes rápidos para sífilis é determinado pelo protocolo clínico de diretrizes terapêuticas, estabelecendo que a gestante deve realizar três testes rápidos: o primeiro no primeiro trimestre de gestação; o segundo no início do terceiro trimestre; e o terceiro no momento do parto ou aborto, independentemente de exames anteriores^24^. Porém, encontramos um valor muito inferior do estabelecido pelos protocolos, chegando a número de testes rápidos quase oito vezes menor que o número mínimo para uma única testagem por nascido vivo.

O último inquérito nacional de saúde encontrou uma taxa de cobertura de exames de sífilis em gestantes de 64,8%^25^, outro estudo utilizando uma base de dados hospitalares evidenciou que nenhuma região do Brasil chegou a 95% de testagem para sífilis na primeira consulta do pré-natal. Ao verificar uma segunda testagem, o valor máximo de cobertura chegou apenas a 56%^20^. Assim, o efeito da realização dos testes rápidos para detecção de sífilis na gestação na APS pode ser ainda maior do que os encontrados, o que poderia indicar uma importância ainda mais evidente do uso de testes rápidos nesses locais para detecção da sífilis durante o pré-natal.

A qualidade da assistência ao pré-natal é um dos fatores importantes para a realização dos testes rápidos na APS, o que é corroborado por um artigo desenvolvido por Mário e colaboradores^26^ investigou a qualidade do pré-natal a partir de um inquérito nacional, apontando que 80,6% das gestantes foram classificadas como tendo um pré-natal adequado e, embora não tenha trabalhado diretamente o efeito do teste rápido para a qualidade do pré-natal, o diagnóstico da sífilis na APS faz parte para classificação de um pré-natal adequado. Dessa forma, podemos levantar a hipótese de que a melhoria da qualidade ao pré-natal nos municípios brasileiros pode ter aumentado o número de testagens em gestantes, principalmente devido ao fortalecimento de programas nacionais de melhoria da saúde à gestante e proteção ao recém-nascido.

Como limitação deste estudo, deve-se ter cautela na interpretação de trabalhos que utilizam dados secundários. Os dados fornecidos pela avaliação externa do PMAQ podem conter vieses, uma vez que se trata de informação autorreferida. Além disso, outras variáveis que não foram testadas poderiam encontrar efeitos preditores diferentes na relação do teste rápido com as taxas de sífilis em gestantes, porém, como este trabalho utilizou bancos de dados nacionais, essas são as informações disponíveis que mais se aproximam de uma relação verdadeira entre a testagem da sífilis e o aumento da sífilis em gestantes no Brasil.

## CONCLUSÕES

É intuitivo pensar que o aumento do teste rápido na APS aumente a capacidade na detecção de sífilis em gestantes, o que foi comprovado por este estudo. Houve uma melhoria substancial na quantidade de testes rápidos disponíveis, além do aumento significativo da realização desses testes nas gestantes, o que prediz o aumento das taxas de sífilis em gestantes.

Porém, diante dos resultados apresentados, podemos aventar que a quantidade de testes realizados em gestantes no período analisado foi insuficiente para detectar o avanço da epidemia nesta população, principalmente pela presença de uma estabilização na quantidade de realização dos testes rápidos e ausência de um platô das taxas de sífilis em gestantes. Essa hipótese é preocupante porque pode indicar que o descontrole da sífilis em gestantes, em parte, se deve à insuficiência no número de testagens para detectar os casos existentes nessa população.

Portanto, investir no aumento da oferta de testes rápidos de sífilis para em gestantes, além de subsidiar estratégias que fortaleça o aumento do número dessas testagens durante o pré-natal, mostra-se como uma estratégia importante no controle da epidemia no país, o que poderia reduzir as taxas de sífilis congênita. O desenvolvimento de novos estudos que busquem avaliar o custo-efetividade dessa medida em âmbito local, comparando com outras estratégias, poderia ampliar os critérios de uso de teste rápido na população gestante.
